# Transcriptional regulation of the operon encoding stress-responsive ECF sigma factor SigH and its anti-sigma factor RshA, and control of its regulatory network in *Corynebacterium glutamicum*

**DOI:** 10.1186/1471-2164-13-445

**Published:** 2012-09-03

**Authors:** Tobias Busche, Radoslav Šilar, Martina Pičmanová, Miroslav Pátek, Jörn Kalinowski

**Affiliations:** 1 Centrum für Biotechnologie, Universität Bielefeld, 33594, Bielefeld, Germany; 2 Institute of Microbiology, Academy of Sciences of the Czech Republic, Vídeňská, 1083, Prague 4, Czech Republic

**Keywords:** *Corynebacterium glutamicum*, ECF sigma factor, Anti-sigma factor, Promoter, Microarray analysis

## Abstract

**Background:**

The expression of genes in *Corynebacterium glutamicum*, a Gram-positive non-pathogenic bacterium used mainly for the industrial production of amino acids, is regulated by seven different sigma factors of RNA polymerase, including the stress-responsive ECF-sigma factor SigH. The *sigH* gene is located in a gene cluster together with the *rshA* gene, putatively encoding an anti-sigma factor. The aim of this study was to analyze the transcriptional regulation of the *sigH* and *rshA* gene cluster and the effects of RshA on the SigH regulon, in order to refine the model describing the role of SigH and RshA during stress response.

**Results:**

Transcription analyses revealed that the *sigH* gene and *rshA* gene are cotranscribed from four *sigH* housekeeping promoters in *C. glutamicum*. In addition, a SigH-controlled *rshA* promoter was found to only drive the transcription of the *rshA* gene. To test the role of the putative anti-sigma factor gene *rshA* under normal growth conditions, a *C. glutamicum rshA* deletion strain was constructed and used for genome-wide transcription profiling with DNA microarrays. In total, 83 genes organized in 61 putative transcriptional units, including those previously detected using *sigH* mutant strains, exhibited increased transcript levels in the *rshA* deletion mutant compared to its parental strain. The genes encoding proteins related to disulphide stress response, heat stress proteins, components of the SOS-response to DNA damage and proteasome components were the most markedly upregulated gene groups. Altogether six SigH-dependent promoters upstream of the identified genes were determined by primer extension and a refined consensus promoter consisting of 45 original promoter sequences was constructed.

**Conclusions:**

The *rshA* gene codes for an anti-sigma factor controlling the function of the stress-responsive sigma factor SigH in *C. glutamicum*. Transcription of *rshA* from a SigH-dependent promoter may serve to quickly shutdown the SigH-dependent stress response after the cells have overcome the stress condition. Here we propose a model of the regulation of oxidative and heat stress response including redox homeostasis by SigH, RshA and the thioredoxin system.

## Background

*Corynebacterium glutamicum* is a gram-positive, non-sporulating soil bacterium that belongs to the order *Actinomycetales,* which also includes genera like *Mycobacterium* and *Streptomyces*. *C. glutamicum* has been studied extensively because of its biotechnological application in the production of various amino acids. Besides this, it is of increasing importance as a model organism for other corynebacteria with biotechnological or medical significance, as well as for the species of related genera [1-3]. The data provided by the complete *C. glutamicum* genome sequence [4-6] enabled genome-wide analyses and the application of comparative genomics to assign functions to uncharacterized genes and to compare the genetic make-up with that of other bacterial species. Although the functions of the genes encoding transcriptional regulators or sigma factors of RNA polymerase may be assigned using comparative genomics, their role and connections in cell regulatory networks could hardly be deduced on the basis of genome sequences alone. Comparative transcriptome analyses of wild-type and mutant strains provide extensive sets of data enabling the connections between the nodes of the regulatory network to be determined.

Transcription initiation, in which an RNA polymerase (RNAP) holoenzyme plays the key role, is a major step in the regulation of bacterial gene expression. The RNAP core enzyme responsible for its catalytic activity consists of five subunits (α^2^ββ`ω) and associates with the σ subunit (factor), which is responsible for specific recognition of the promoter, to complete the fully functional RNAP holoenzyme. The majority of bacteria possess several sigma factors, which direct RNAP to different groups of promoters. The sigma factors thus form a specific class of regulators, which may affect the expression of large gene groups.

σ^70^-family sigma factors are categorized into four different classes [7]. The essential (primary) group 1 sigma factors are responsible for the transcription of housekeeping genes, group 2 contains the primary-like sigma factors, group 3 sigma factors control genes involved in specific functions in some bacteria and group 4 sigma factors (also called ECF for extracytoplasmic function) are involved in responses to external stresses.

In *C. glutamicum,* SigA, the primary sigma factor (group 1), SigB, a primary-like sigma factor (group 2), and SigC, SigD, SigE, SigH and SigM, all of them ECF-type sigma factors, were found [8]. SigB, SigE, SigH, and SigM are the only *C. glutamicum* sigma factors that have been studied so far. The genes included in their regulons were found to be involved in various stress responses [9-12].

Sigma factors are controlled by modulating their availability and activity. Anti-sigma factors bind to their cognate sigma factors in some cases, inhibiting their binding to the RNAP core enzyme. Controlling their activity by the reversible binding of an anti-sigma factor to the sigma factor in *C. glutamicum* was up to now only described for SigE by CseE [10]. The activity of SigH or its orthologs is tightly controlled by anti-sigma factors in various actinobacteria. This has been demonstrated for *M*. *tuberculosis* RshA (a regulator of SigH) and *S*. *coelicolor* RsrA (a regulator of SigR, a SigH ortholog) that bind to their cognate sigma factors in a redox-responsive manner [11,12]. Upon the oxidation of specific cysteine residues these anti-sigma factors change conformation, the respective bound sigma factor is released and can thus bind to RNAP, thereby activating its sigmulon (regulon of a sigma factor). After the cessation of the oxidative stress conditions, the reduced state is regenerated by the action of thioredoxins, and the anti-sigma factors regain their SigH-binding ability. The conserved cysteine residues have a conserved arrangement, the ZAS (zinc-containing anti-sigma factor) domain and the anti-sigma factors from different organisms can functionally replace each other [13].

It has been shown that *C. glutamicum* SigH is involved in responses to heat shock [14] and oxidative stress [15]. The crucial role of SigH in the heat-shock response by controlling the expression of the ATP-dependent Clp protease, chaperones and heat-shock regulators was demonstrated in a number of studies [14-18]. The SigH-driven response to oxidative stress in actinobacteria generally includes the upregulation of the thioredoxin system (*trxB* and *trxC*) and at least one gene (*mtr*) of the mycothiol system, which are major antioxidant systems in these bacteria [19].

In addition to its involvement in the expression of a number of heat-shock response genes, *C. glutamicum* SigH was found to control the expression of genes encoding various stress regulators, such as HspR [18], ClgR [16], SufR [14], WhcA [20] and WhcE [21]. Moreover, transcription of the genes encoding the sigma factors SigB and SigM is controlled by SigH [22-24]. Since SigH was found to be a major player in response to heat shock and oxidative stress, a regulatory network integrating the sigma factors SigH, SigB and SigM is apparently operative in *C. glutamicum*.

In this work, we demonstrate that the genes *sigH* and *rshA,* coding for the stress-responsive sigma factor and its putative anti-sigma factor, respectively, form an operon in *C. glutamicum* and are transcribed from multiple promoters of different classes. The SigH-dependent genes were defined on the basis of their enhanced transcription in the Δ*rshA* strain in the absence of environmental stimuli by DNA-microarray analysis and by q-RT-PCR*.* These results validated the assumption that RshA acts as an anti-SigH factor. We propose a model of the SigH-RshA regulatory network underlining the central role of SigH in the stress response of *C. glutamicum.*

## Results

### The *sigH gene* and the *rshA* gene encoding an anti-sigma factor of SigH form an operon

**Figure 1:**
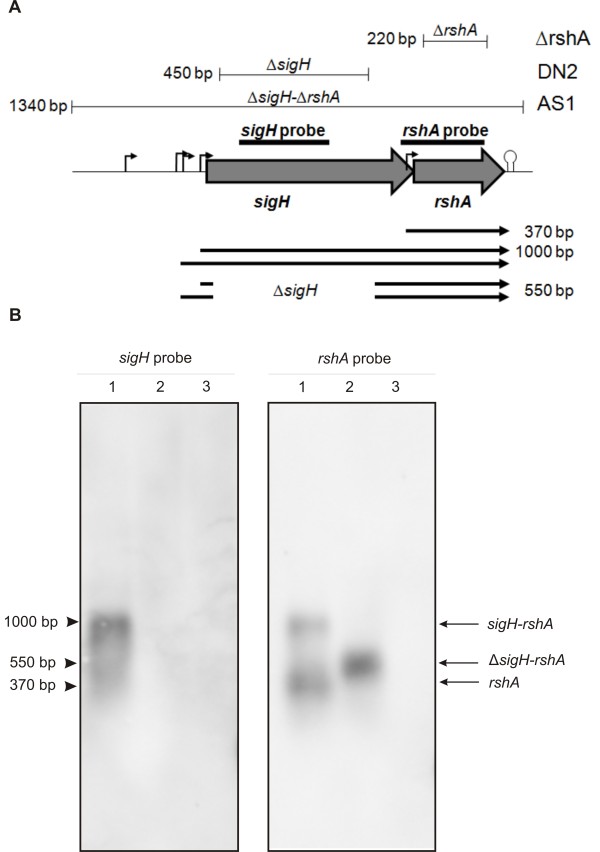
**Genetic map of the *sigH-rshA *operon, its Northern hybridization analysis in *C. glutamicum *RES167 and its deletion derivatives. A**. Genetic map of the *sigH-rshA* region showing locations and sizes of deletions in the chromosomes of strains *C. glutamicum* Δ*rshA*, DN2 and AS1, predicted sizes of respective *sigH-rshA* and *rshA* transcripts (arrows) and locations of probes used for Northern hybridizations. Promoters are indicated with bent arrows and the terminator with a hairpin symbol. **B**. Northern blot using a *sigH* probe (left panel) and an *rshA* probe (right panel) hybridized with total RNA extracted from: RES167 cells (lane 1); DN2 cells (Δ*sigH* deletion; lane 2); AS1 cells (Δ*sigHrshA* deletion; lane 3). The estimated lenghts of the detected transcripts (left) and their designations (right) are indicated. The sizes of the fragments in the RNA marker are indicated with arrows.

The genes encoding SigR (an ortholog of *C. glutamicum* SigH) in *S. coelicolor* and SigH in some mycobacteria (e.g. *M. smegmatis* and *M. avium*) are located in close proximity to the genes encoding their anti-sigma factors RsrA and RshA, respectively, which were found immediately downstream [12,25]. The same arrangement of the *sigH* (*cg0876*) and *rshA* (*cg0877*) genes was described in the genomes of *C. glutamicum* ATCC 13032 [5] and *C. jeikeium*[26] (Figure 1a). Probably due to its small size of 267 nucleotides (89 amino acids), the *rshA* gene has not been annotated in two other *C. glutamicum* genome sequences, but it can also be found there by using a BLASTX search (data not shown). It is interesting to note that the absence of the *rshA* gene in the annotation of one of the *C. glutamicum* genome sequences [4] apparently misled the authors of a recent study [13], who picked the wrong ortholog from *C. glutamicum* in order to check for a functional complementation of *rsrA* in *Streptomyces*. It is not surprising that the above-mentioned study failed to show a functional complementation.

In all *C. glutamicum* genomes, the translational stop codon of *sigH* is only separated by two bp from the translation initiation codon of *rshA*, indicating an operon-like structure. The deduced RshA protein sequence from *C. glutamicum* is only moderately similar to that of RshA from *M*. *tuberculosis* (35%) and RsrA from *S*. *coelicolor* (28%). An amino acid sequence alignment between the three corynebacterial genes and their *M*. *tuberculosis* and *S*. *coelicolor* counterparts (Additional file 1) shows that RshA from *C. glutamicum* carries the conserved cysteine residues which mediate the interaction of SigH and RshA in the ZAS domain [13].

The *sigH* gene and the *rshA* gene form an operon-like structure in *C. glutamicum*. We therefore first analyzed their transcriptional organization by Northern hybridization. The blotting was performed with total RNA prepared from *C. glutamicum* RES167 (restriction-deficient variant derived from the ATCC 13032 type strain and its derived deletion mutant strains DN2 (carrying a deletion within *sigH*) and AS1 (carrying a complete deletion of *sigHrshA*). The blot was then hybridized with DIG-labelled RNA-probes derived from the *sigH* and the *rshA* genes, respectively. A single 1-kb transcript hybridized with the *sigH* riboprobe when total RNA isolated from the RES167 strain was used (Figure 1b). A transcript of the same length also hybridized with the *rshA* riboprobe. These results indicated that both genes are transcribed in a single mRNA from a promoter located upstream of the *sigH* gene. An additional transcript of approximately 370 bp was detected by using the *rshA* riboprobe. This transcript most likely only covered the *rshA* gene and suggested that another promoter (P*rshA*) is present within the *sigH* coding region.

To address the question of whether the promoters of the *sigH* and *rshA* genes are controlled by the sigma factor SigH, we used RNA isolated from the *sigH* deletion strain DN2 for Northern hybridization. We supposed that the SigH*-*dependent transcripts would not be found with DN2 RNA. Indeed, no signal was detected when the *sigH* probe was used, because the complementary region in the *sigH* gene was deleted in DN2. A transcript of around 550 bp was detected with the *rshA*-specific probe (Figure 1b). This transcript most probably initiated upstream of *sigH* (from the *sigH* promoter), since its length was that of the full-length transcript containing *sigH-rshA* minus the length of the deletion within *sigH* in DN2 (Figure 1a). These results suggested that the bicistronic *sigH-rshA* transcript is formed in a SigH-independent manner. In contrast, the *rshA* transcript was not detected with the *rshA* probe, although the deletion within *sigH* should not have removed the presumed *rshA* promoter. This result indicated that the *rshA* promoter is under the control of SigH.

### Genes of the *sigH-rshA* operon are transcribed from multiple promoters of different types

To analyze the promoter regions of the *sigH*-*rshA* operon and of the *rshA* gene, DNA fragments (504 bp upstream of *sigH* and 301 bp upstream of *rshA*) were cloned in the promoter probe vector pET2, thus forming transcriptional fusions of the promoter-active fragments and the reporter gene *cat* coding for chloramphenicol acetyltransferase (CAT). The activity of the promoters was measured using the CAT enzyme activity in cell-free extracts of *C. glutamicum* (pET2*sigH*) and *C. glutamicum* (pET2*rshA*). The activity of P*sigH* during the exponential growth phase was 0.1±0.015 U (mg of protein)^-1^ whereas the activity of P*rshA* was only 0.03 ±0.005 U (mg of protein)^-1^. Negligible activity was detected with the empty vector pET2 (≤0.003 U (mg of protein)^-1^). These measurements confirmed that *rshA* is also transcribed from the separate P*rshA* promoter.

**Figure 2:**
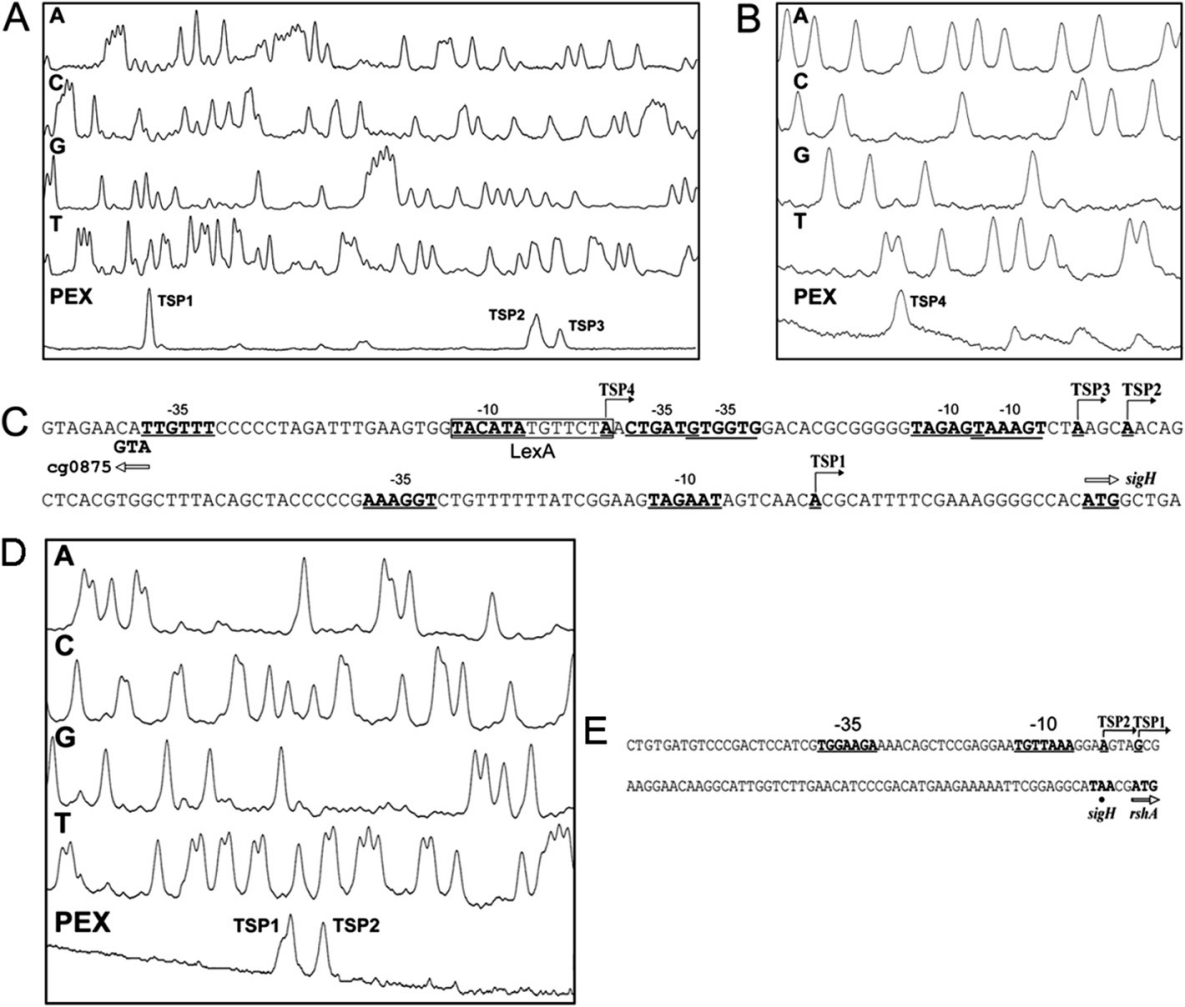
**Determination of transcriptional start points of the *sigH *and *rshA *genes and sequences of their promoter regions. (a)** and **(b)** Determination of the *sigH* transcription start sites (TSP) by primer extension analysis. The bottom peaks (PEX) represent cDNA synthesized in the reverse transcription using RNA from *C. glutamicum* (pET2sigH) and *C. glutamicum* (pET2*sigH*4), respectively. The smaller peaks were not reproducibly observed in the repeated experiments. The peaks (A, C, G, T) represent the products of sequencing reactions carried out with the same fluorescent-labeled primer as that used for reverse transcription. **(c)** Nucleotide sequence of the *sigH* upstream region. TSPs and the proposed −35 and −10 promoter elements are in bold and underlined. Transcription initiation is indicated by the bent arrows. The proposed binding site for the LexA regulator is boxed and the initiation codons (in bold) of the genes *sigH* and cg0875 are indicated with hollow arrows. **(d)** Determination of *rshA* TSP. **(e)** Nucleotide sequence of the *rshA* upstream region. The stop codon (in bold) of *sigH* is indicated with the black dot. Note that the sequences **(c)** and **(e)** are complementary and reversed to those deduced from the peaks generated by the sequencer.

To determine the transcriptional start points (TSPs) of the *sigH-rshA* and *rshA* transcripts, a primer extension analysis was performed (PEX) using the primer CM4 and total RNA isolated from *C. glutamicum* (pET2*sigH*) and *C. glutamicum* (pET2*rshA*), respectively. Three TSPs were located within the upstream region of the *sigH* gene. TSP1, TSP2 and TSP3 were mapped at nucleotide A in all cases, 22 nt, 89 nt and 93 nt upstream of the *sigH* start codon, respectively (Figure 2a). An identical result was achieved with the primer CM5 (data not shown). The putative −10 hexamers of the respective promoters, TAGAAT (P1), TAAAGT (P2) and TAGAGT (P3) are similar to each other and fit well to the consensus −10 hexamer TANANT of SigA-dependent promoters driving the expression of housekeeping genes in *C. glutamicum*[8]. The putative −35 sequences of P1, P2 and P3 are less similar to the consensus, which is a common feature of *C. glutamicum* housekeeping promoters. In conclusion, all three promoters seem to be SigA-dependent. Since yet another TSP signal could be recognized further upstream of TSP3 in some primer extension analyses using *C. glutamicum* (pET2*sigH*), the 348-bp upstream fragment (462 to 115 nt upstream of the *sigH* initiation codon, outside of P1, P2 and P3) was separately cloned in pET2 (resulting in pET2*sigH*4). Using this transcriptional fusion, a CAT activity of 0.009±0.002 U (mg of protein)^-1^ was determined. This result indicated that there is a promoter within this upstream fragment. With RNA isolated from *C. glutamicum* (pET2s*igH*4) and primers CM4 or CM5, transcription start point at nt A, (TSP4) 131 nt upstream of the *sigH* initiation codon was determined by PEX (Figure 2b). The position of TSP4 was further confirmed by RACE analysis (data not shown). The hexamer TACATA located the appropriate distance from TSP4 and the hexamer TTGTTT (with a spacer of 19 nt) could function as the −10 and −35 sequences of another SigA-dependent promoter (P4), respectively (Figure 2c). A TGGTACATATGTTCTA sequence conforming to the consensus sequence of the SOS box, which was described as a LexA binding site in *C. glutamicum*[27], was found to overlap with the −10 region of P4.

Using total RNA from *C. glutamicum* (pET2*rshA*) and the CM4 primer, two TSPs were detected at nt G and A, 62 nt and 66 nt upstream of the *rshA* initiation codon (Figure 2d). TSP1 at the same G was detected by a weaker PEX result with the CM5 primer (not shown). The motifs TGGAAGA in the −35 region and TGTTAAA in the −10 region relative to TSP1 fit well to the consensus sequence of the −35 and −10 regions of the proposed SigH-dependent promoters of the *M*. **tuberculosis** (^G^/T**GGAA**^C^/TA −16 nt –^C^/G**GTT**) [28] and SigR-dependent promoters of *S. coelicolor* (G**GGAA**T^G^/C - 16 nt - ^C^/G**GTT**G) [29] and also to the proposed *C. glutamicum* consensus of SigH-dependent promoters g**GGAA**ta - 16–19 nt - ^C^/T**GTT**gaa [14] or ^G^/T**GGAA**TA - 16–19 nt - ^C^/T**GTT**GAA [8]. This result suggests that the P*rshA* promoter is under the control of SigH, which is in agreement with the results from the Northern hybridization experiments.

### Global transcriptional profiling of the *rshA* deletion mutant revealed the majority of known SigH-dependent genes and novel ones

**Figure 3:**
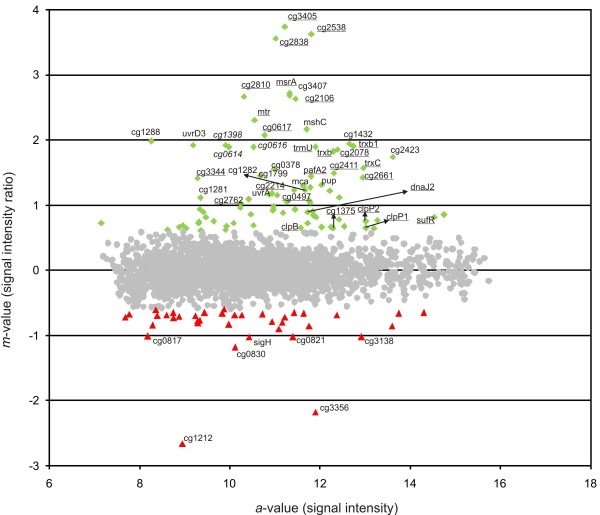
**Microarray analysis of the *C. glutamicum *RES167 strain compared with its Δ *rshA *mutant DN2. **Ratio/intensity plot obtained from the DNA microarray comparing the transcriptomes of RES167 and DN2 is shown. Total RNA was isolated from two biological replicates grown in minimal CGXII medium to the exponential phase and used for hybridization. Genes with increased amounts of mRNA in the Δ*rshA* strain have positive ratios, while genes with a higher mRNA amount in the RES167 strain have negative ratios, indicated with green diamonds (upregulated) or red triangles (downregulated) respectively; those not exhibiting differential expression are indicated with grey spots. M values of higher than +0.6 or lower than −0.6 (corresponding to fold changes of 1.52 and 0.66, respectively) were considered to be significant. The relevant genes are indicated by their names or desigations from the *C. glutamicum* genome sequence (GenBank NC_006958), underlined genes were previously described as SigH-dependent.

To discover genes that are under the control of SigH, we utilised the constructed *C. glutamicum* Δ*rshA* strain. We expected that SigH would be released from inhibition by the anti-sigma factor in this deletion strain and SigH-dependent genes might be expressed without applying any stress. A comparative microarray hybridization analysis was performed using total RNA isolated from *C. glutamicum* RES167 and its *rshA* deletion derivative growing under standard cultivation conditions (30°C) in shaking flasks. The signal intensity ratio (m) / signal intensity (a) plots deduced from hybridizations are shown in Figure 3 and the differentially transcribed genes are listed in Table 1. Altogether, 83 genes in 61 putative transcriptional units were found to be upregulated in the Δ*rshA* mutant compared to its parent strain. The highest ratios were observed for the genes previously described as members of the SigH regulon [14]. These data strongly confirmed the assumption that the SigH sigma factor would be highly active in the Δ*rshA* strain in which the functional *rshA* gene product is absent and are in line with the notion that RshA plays the role of an anti-sigma factor controlling SigH activity *in vivo*.

**Table 1:** **Genes with enhanced expression in *C. glutamicum *Δ*rshA *compared with *C*. *glutamicum *RES167 (reference) sorted by function**

***Coding sequence***^***a***^	***Gene***	**Predicted function**	***Fold change***^**b**^
***Disulphide stress related genes***			
cg3405◊*		NADPH:quinone reductase	13.27
cg2538		Alkanal monooxygenase (FMN-linked)	12.3
cg2838		Putative dithiol-disulfide isomerase	11.71
cg3236	*msrA*	Protein-methionine-S-oxide reductase	6.59
cg2194	*mtr*	Putative NADPH-dependent mycothiol reductase	4.92
cg1709▫^-^	*mshC*	Putative 1-D-myo-inosityl-2-amino-2-deoxy-alpha- D-glucopyranoside—L-cysteine ligase	4.47
cg3299	*trxB1*	Thioredoxin (TRX)	3.73
cg2078	*msrB*	Peptide methionine sulfoxide reductase	3.61
cg3422○*	*trxB*	Thioredoxin reductase	3.53
cg3423○	*trxC*	Thioredoxin	2.97
cg2661		Putative dithiol-disulfide isomerase	2.68
cg3344		Putative nitroreductase	2.66
cg1127▪	*mca*	Putative mycothiol S-conjugate amidase	2.46
cg2214		Putative Fe-S-cluster redox enzyme	2.27
cg0497□*	*mca*	Glutamyl-tRNA reductase	2.07
cg1765	*sufR*	Transcriptional repressor of suf operon	1.75
cg1553	*qor2*	quinone oxidoreductase involved in disulfide stress response	1.60
cg1375		Putative thioredoxin	1.58
			1
***Heat stress-related genes***			
cg2515	*dnaJ2*	Chaperone, contains C-terminal Zn-finger domain	1.85
cg2644:	*clpP2*	Endopeptidase Clp, proteolytic subunit	1.68
cg3079●*	*clpB*	Putative ATP-dependent protease (heat-shock protein)	1.57
cg2645:*	*clpP1*	Endopeptidase Clp, proteolytic subunit	1.57
***SOS and DNA repair genes***			
cg1555	*uvrD3*	DNA/RNA helicase, superfamily I	3.78
cg1560	*uvrA*	Excinuclease ABC, ATPase subunit A	2.23
cg0184^*		Putative RNA-binding protein	1.95
cg0185^		Putative glyoxalase	1.91
cg0186^		Putative methylated-DNA--protein-cysteine methyltransferase	1.91
cg1795	*uvrC*	Excinuclease subunit C	1.53
***Proteasome components***			
cg1688°::	*pafA2*	Putative proteasome component	2.71
cg1689°^::^*	*pup*	prokaryotic ubiquitin-like protein	2.48
cg0998		Trypsin-like serine protease	1.54
***Genes with other function***			
cg3407◊		Putative membrane protein	6.41
cg2106		Conserved hypothetical protein	6.19
cg0617†*		Hypothetical protein	4.20
cg1288		Putative multidrug efflux permease, MFS-type	3.94
cg1432	*ilvD*	Dihydroxy-acid dehydratase	3.84
cg1398‡		Conserved hypothetical protein	3.78
cg0614†		Hypothetical protein	3.71
cg0616†	*fdhD*	Putative formate dehydrogenase, FdhD-family	3.71
cg1397‡*	*trmU*	tRNA (5-methylaminomethyl-2-thiouridylate) -methyltransferase	3.71
cg2423	*lipA*	Lipoyl synthetase	3.34
cg0378		Putative phage-associated protein	2.93
cg2411		Conserved hypothetical protein, HesB/YadR/YfhF family	2.81
cg1799·*	*ribC*	Riboflavin synthase, alpha chain	2.73
cg2247		Hypothetical protein	2.41
cg1282		Conserved hypothetical protein	2.35
cg2127		Hypothetical protein	2.35
cg3424○	*cwlM*	N-Acetylmuramoyl-L-alanine amidase	2.33
cg1798·	*ribA*	Putative GTP cyclohydrolase II/3,4-dihydroxy-2-butanone-4-phosphatesynthase	2.22
cg2835		Putative acetyltransferase	2.17
cg1281		ABC-type putative multidrug transporter, ATPase and permease subunit	2.16
cg1687°^::^		Putative transcriptional regulatory protein	2.13
cg1797·	*ribH*	Riboflavin synthase, beta chain	2.10
cg1779	*opcA*	Glucose-6-phosphate 1-dehydrogenase subunit	2.06
cg2762	*murI*	Glutamate racemase	2.03
cg3078●		Hypothetical protein	1.97
cg1411°	*rbsA*	ABC-type ribose transporter, ATPase subunit (TC 3.A.1.2.1)	1.92
cg2636	*catA*	Catechol 1,2-dioxygenase	1.88
cg1780	*pgl*	6-Phosphogluconolactonase	1.87
cg1413°	*rbsB*	ABC-type ribose transporter, substrate-binding lipoprotein (TC 3.A.1.2.1)	1.85
cg0498□	*hemC*	Porphobilinogen deaminase	1.84
cg2665		Hypothetical protein	1.82
cg2181†*		ABC-type putative dipeptide/oligopeptide transporter, substrate-binding lipoprotein	1.80
cg1128▪		Hypothetical protein, similar to ribosomal protein S2	1.79
cg1139		Allophanate hydrolase subunit 2	1.75
cg1708^-^		Conserved hypothetical protein	1.75
cg2560	*aceA*	Isocitrate lyase	1.72
cg2183†		ABC-type putative dipeptide/oligopeptide transporter, permease subunit	1.71
cg2434		Putative monooxygenase, luciferase	1.68
cg0380		Hypothetical protein	1.67
cg0043		ABC-type putative manganese/zinc transporter, ATPase subunit	1.65
cg0228		Two-component system, sensory histidine kinase, putative pseudogene	1.65
cg1412°	*rbsC*	ABC-type ribose transporter, permease subunit (TC 3.A.1.2.1)	1.65
cg1778	*zwf*	Glucose-6-phosphate 1-dehydrogenase	1.65
cg1686°		Putative transcriptional regulatory protein	1.61
cg1482		Putative Zn-dependent hydrolase	1.61
cg2514		Conserved hypothetical protein	1.59
cg2206	*ispG*	4-hydroxy-3-methylbut-2-en-1-yl diphosphate synthase	1.58
cg2546		Putative secondary C4-dicarboxylate transporter, tripartite ATP-independent transporter (TRAP-T) family	1.58
cg0699	*guaB2*	IMP dehydrogenase	1.56
cg2184		ABC-type putative dipeptide/oligopeptide transporter, ATPase subunit	1.56
cg3077●		Putative membrane protein	1.56
cg1410°*	*rbsR*	Transcriptional repressor of ribose importer RbsACBD, LacI-family	1.54
cg1464		Putative transcriptional regulator, HTH_3-family	1.52

^*a*^Genes constituting a putative operon are indicated with the same symbol. The first gene in the operon is indicated with an asterisk. Underlined genes were previously described as SigH-dependent [14,16].

^*b*^*fold change*, signal intensity ratio as defined by 2^(m-value)^.

Although most of the differentially transcribed genes match those described by Ehira *et al.*[14], this study also found the genes *mshC* (*cg1709;* mycothiol synthesis) and *mca* (*cg1127,* mycothiol conjugate amidase) to be strongly deregulated, and *qor2* (*cg1553,* quinone oxidoreductase) as weakly influenced in the Δ*rshA* mutant. All of these genes are apparently involved in redox homeostasis and were also found to be more strongly transcribed under disulphide stress conditions induced by diamide treatment (our unpublished results).

Interestingly, some heat-stress related genes previously reported to be SigH-dependent (*dnaJ2, clpB, clpP1* and *clpP2;*[14,16]) showed up only weakly in our analyses and some other previously identified members of the SigH regulon failed to exhibit the minimum threshold (m-value of 0.6 corresponding to 1.5-fold change) used. Genes that displayed differential expression values below this threshold were the *dnaK-grpE* operon, *clpC,* the non-essential sigma factor gene *sigB* and most genes of the *suf* cluster [14]. A differential transcription of *clgR*, a heat stress-responsive regulator, which was expressed from a SigH-dependent promoter accoding to Engels *et al.*[16], was also not detected in our experiments. This finding is similar to observations by Ehira *et al.*[14]). These discrepancies might be explained by additional regulatory systems negatively controlling the transcription of these genes in the absence of (heat) stress.

Genes identified for the first time as being triggered by the SigH-RshA regulatory network included *uvrA* (*cg1560*) and *uvrC* (*cg1790*)*,* both coding for subunits of the Exinuclease ABC (nucleotide-excision repair), as well as *uvrD3* (*cg1555*), one of three genes encoding DNA helicases similar to UvrD proteins in *C. glutamicum*, and a gene cluster (*cg0184*-*cg0186*) possibly involved in alkylated DNA repair. Together with the observation of a putative LexA-regulated promoter upstream of the *sigH-rshA* operon, this links the SigH network with DNA damage and repair.

Other newly identified genes code for components of the proteasome machinery, *pup* (*cg1689*; encoding a prokaryotic ubiquitin-like protein) and *cg0998* (a trypsin-like serine protease). All these genes were found to be transcriptionally induced in the Δ*rshA* strain (Table 1).

Among the downregulated genes, only 7 exceeded the standard threshold m < −1 (fold change 0.5). These genes encode putative membrane proteins, hypothetical proteins and transporters (Additional file 2). Interestingly, the *sigH* transcript itself appeared to be less abundant in the *rshA* deletion mutant. Since this result was unexpected, we checked P*sigH* for mutations in this strain by PCR amplification and sequencing of the *sigH* 5’-upstream region. No mutations were found within 315 bp upstream of the *sigH* translational start codon (data not shown). It can be speculated that the *sigH* transcript is less stable in the Δ*rshA* mutant due to a change in its structure or due to the lack of stablisation effects by ribosomes translating *rshA*.

To validate the newly found potential SigH-dependent genes, we focussed our subsequent analyses on those from which new insights into the SigH regulon were expected. Therefore the genes potentially involved in response to disulphide stress, in protein degradation and in SOS response to DNA damage were included in the following q-RT-PCR experiments.

### Differential transcription of selected SigH-dependent genes was validated by quantitative real-time RT-PCR

The microarray analyses found a number of novel candidate genes for the SigH regulon. To validate these results, we performed a q-RT-PCR with *mshC*, *mca* and *mtr* (involved in mycothiol synthesis and recycling [30,31]), *pup* (encoding an ortholog of the recently identified prokaryotic ubiquitin-like protein in *M*. *tuberculosis*[32]), as well as *uvrA* and *uvrD3* (SOS-response). Additionally, we chose the two genes with strongly enhanced expression in the Δ*rshA* strain, *cg2838* (putative dithiol-disulfide isomerase) and *cg3405* (NADPH:quinone reductase), which might be involved in defense against disulphide stress. The recently described small antisense RNA *arnA* that has been shown to be transcribed from a SigH-dependent, heat-shock-induced promoter [33] was also included in the q-RT-PCR analysis. The *arnA* transcript was not addressed in the microarray analysis, since only probes for protein-coding genes were used in the design of the microarray [34].

**Figure 4:**
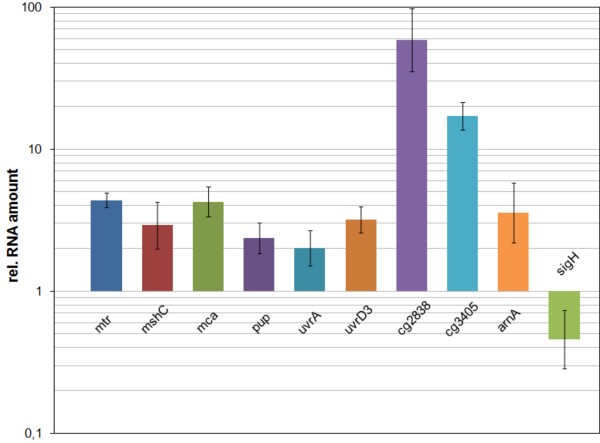
**Relative transcript levels of selected potential SigH-dependent genes in *C. glutamicum *Δ *rshA/C. glutamicum *RES167 measured by q-RT-PCR. **The data obtained for the RES167 strain served as a reference and the respective values were set to 1.0 on the logarithmic scale. Three biological replicates for the Δ*rshA* strain and four replicates for the RES167 strain were analysed in duplicate. SD values are shown as error bars.

The strong transcriptional induction observed in microarray analysis was validated for both *cg2838* and *cg3405* with 60-fold and 20-fold higher transcript levels, respectively (Figure 4). The genes *mshC*, *mca*, *mtr*, *uvrD3*, and *arnA*, were induced 3- to 4-fold and the weakest induction was observed for *pup* and *uvrA* with a 2-fold higher transcript level in the Δ*rshA* mutant than in the WT-strain. The reduction of the transcript level of *sigH* to around 50% of the WT level was also confirmed.

### Experimental localization of SigH-dependent promoters and derivation of a consensus sequence

**Figure 5:**
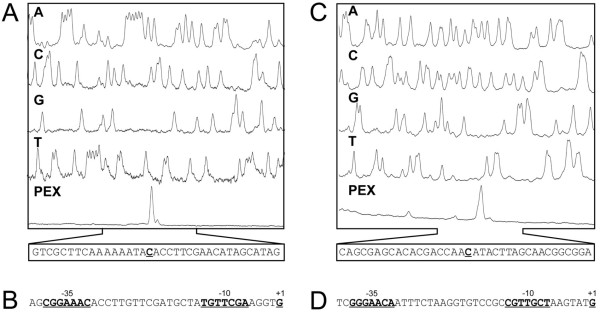
**Determination of transcriptional start points of *uvrA *and *dnaJ2 *genes. (a)** Determination of *uvrA* TSP. A portion of the nucleotide sequence derived from the sequencing peaks is shown below, TSP is in bold and underlined. **(b)***uvrA* promoter sequence. TSP (+1) and the proposed −35 and−10 promoter elements are in bold and underlined. **(c)** Determination of *dnaJ2* TSP. **(d)***dnaJ2* promoter sequence. Note that the sequences at **(b)** and **(d)** are reversed and complementary to those shown in **(a)** and **(c)**.

**Figure 6:**
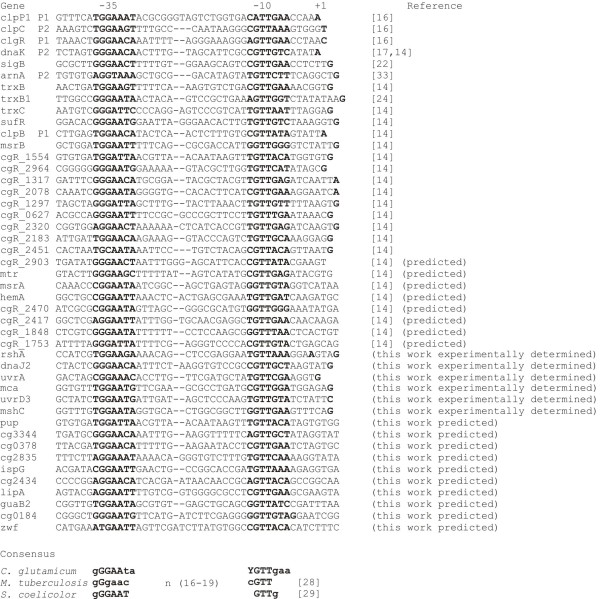
**Sequences of presumed *C. glutamicum *SigH-dependent promoters. **Putative −10 and −35 regions (a spacer of 16 -19 nucleotides) and TSPs (+1) are highlighted in bold. Dashes indicate gaps introduced to align the −35 element. Positions in the *C. glutamicum* consensus with a single nucleotide occurrence of over 80% are in bold letters, K= G or T; Y= C or T; R= A or G;W = A or T. The sequence reported by Halgasova *et al.*[22] is from *C. glutamicum* CCM251, the sequences reported by Ehira *et al.*[14] are from *C. glutamicum* R, and the others are from *C. glutamicum* ATCC 13032.

Several genes which exhibited higher transcript levels in the Δ*rshA* strain than in its parental WT strain in microarray analyses and/or in q-RT-PCR were chosen for promoter localization by TSP determination using primer extension analysis. Regions (300 to 400 bp) upstream of the initiation codons of the analyzed genes were used to construct transcriptional fusions with the *cat* gene in the vector pET2. TSPs within the *mshC*, *mca, dnaJ2*, *uvrA* and *uvrD3* upstream fragments (carrying potential SigH-dependent promoters) were localized 141 nt, 207 nt, 100 nt, 46 nt and 56 nt upstream of the initiation codons, respectively. Examples of the results of primer extension analysis for *dnaJ2* and *uvrA* are shown in Figure 5. The respective −10 and −35 regions which were compatible with the consensus sequence of the SigH-dependent promoters [8,14] were found at the proper distances in all cases (Figure 6). In addition, transcriptional starts within *mca* and *pup* fragments and the respective sequence motifs resembling SigA-dependent promoters were localized upstream of these genes by primer extension (data not shown).

**Figure 7:**
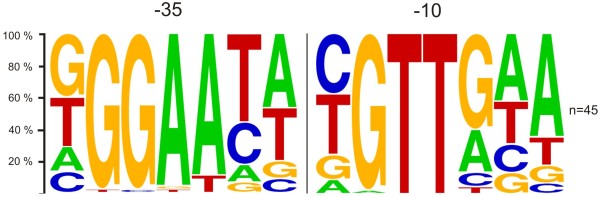
**Distribution of nucleotides within the −35 and −10 core regions of *C. glutamicum *SigH-dependent promoters. **The percentage occurrence of a nucleotide at a particular position is represented by the size of the nucleotide symbol (A, C, G, T) using Weblogo [57]. This analysis is based on 45 presumed SigH-dependent promoters.

Further SigH-dependent promoters were searched for within the 5´-UTRs of the genes, which exhibited enhanced transcription in the Δ*rshA* strain in the microarray analyses, by motif searches using the program Bioprospector [35]. In addition to all previously defined promoters belonging to the genes of the SigH regulon [14], the promoter of *arnA*[33] and the promoters determined in this work (*rshA*, *mshC*, *mca, dnaJ2*, *uvrA* and *uvrD3*) were included in the training set. We searched for two 10-bp motifs with a gap of 15 to 23 bp. Using the Bioprospector program, 10 additional transcriptional units containing a conserved SigH-dependent promoter motif in their 5´-UTR were identified (Figure 6). The other 26 analyzed transcriptional units did not show up in these analyses. Their transcription initiation is possibly not directly SigH-dependent but rather upregulated by secondary effects under the conditions used. Six SigH-dependent promoters upstream of the identified genes were precisely localized by determination of the respective transcriptional start points. A refined consensus sequence based on the sequences of 45 SigH-dependent promoters was defined (Figure 7).

## Discussion

### The *sigH-rshA* operon in *C. glutamicum* exhibits complex transcriptional organization including autoregulation

In this study we demonstrated the upregulation of the majority of the known SigH-dependent genes in the absence of an applied stress by removing its putative anti-sigma factor RshA. The gene encoding RshA was only annotated in the genome of *C. glutamicum* ATCC 13032, reported by Kalinowski *et al.*[5]. The *rshA* gene in two other sequenced *C. glutamicum* strains, in *C. glutamicum* ATCC 13032, reported by Ikeda and Nakagawa [4], and *C. glutamicum* strain R, reported by Yukawa *et al.*[6], is not annotated, probably because of its small size of 89 amino acids. However, the deduced RshA protein sequences are identical in the three genome sequences and similar to other anti-sigma factors from *M*. *tuberculosis* (RshA; [12]) or *S. coelicolor* (RsrA; [11]). RshA from *C. glutamicum* shares the conserved cysteine residues in the ZAS domain with its counterparts. These residues modulate the interaction with the SigH protein, a fact that has been experimentally determined for RshA and SigH in *M*. *tuberculosis*[12], RsrA and SigR in *S. coelicolor*[11], as well as for other members of the ZAS-domain containing protein family in actinobacteria [13]. The clear upregulation of all previously determined SigH-dependent genes in the constructed *rshA* mutant provides further proof that in *C. glutamicum*, RshA functions as an anti-sigma factor similar to *M*. *tuberculosis* RshA and *S. coelicolor* RsrA.

The *sigH-rshA* gene organization is also conserved in all sequenced *Corynebacterium* strains available in NCBI database e.g. *C. glutamicum, C. efficiens, C. jeikum*[8]*,* and in the more distantly related *S. coelicolor. M. tuberculosis* exhibits a similar organization, but a gene encoding a protein of unknown function is inserted between the *sigH* and *rshA* genes.

The transcriptional organization of the *sigH-rshA* operon in *C. glutamicum* is similar but not identical to that of *M*. *tuberculosis* and *S. coelicolor*. In *C. glutamicum*, four promoters upstream of *sigH-rshA* resemble house-keeping promoters which are recognized by SigA. The reason for having multiple promoters might ensure fine-tuning, either by the action of additional transcription factors or by the differing affinities of these promoters to SigA and SigB, the non-essential sigma factor of *C. glutamicum* that also targets house-keeping promoters [9]. Experimental observations are in line with this assumption: it was shown by a reporter fusion analysis (P-*sigH*::*cat*) that the activity of the *sigH* promoter rose in the stationary phase and after oxidative stress [15], whereas no significant changes in *sigH* transcript levels were detected after heat shock [18] or in the transition phase of growth [24]. In *S. coelicolor*, the *sigR-rsrA* operon is also transcribed from multiple promoters. There is one transcriptional start of *sigR* dependent on the housekeeping sigma factor SigA and another one dependent on SigR^Sc^ itself [25]. In *M. tuberculosis*, *sigH* is apparently only autoregulated by SigH [12].

A possible additional regulation of SigH in *C. glutamicum* might operate via the SigA-dependent promoter that was found in the 5´-UTR of the *sigH* gene, overlapping with a putative SOS-box [27] and therefore most likely blocked by the LexA repressor in the absence of a DNA-damaging agent.

The main difference from the related bacteria *S. coelicolor* and *M*. *tuberculosis* was the finding that in *C. glutamicum*, the *rshA* gene is transcribed by an additional promoter as a monocistronic transcript. We showed by Northern blotting and by PEX analysis that this transcription is SigH-dependent. It can be speculated that this transcriptional organization evolved to guarantee an excess of RshA protein over SigH at all times and therefore a fast shut-down of SigH-dependent transcriptional activation as soon as stress conditions end.

### Expression analysis of the *rshA* mutant strain validated and extended the known SigH regulatory network

**Figure 8:**
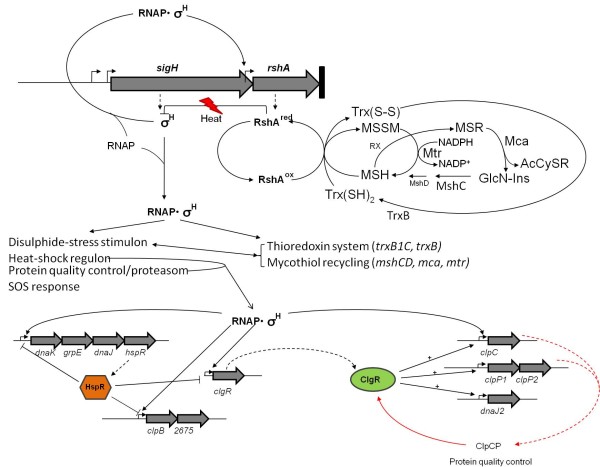
**Extended model of the SigH regulatory network in *C. glutamicum. ***Conditions that deplete thiols by oxidation or alkylation cause the oxidation of cysteine residues inside RsrA. RsrA_ox_ is released from SigH, which then binds to the RNA polymerase (RNAP) and initiates the transcription of its regulon. Direct induction of *trxBC/B1, mca*, *mshC* and *mtr* genes that are involved in the disulphide response generate and recycle/reduce the thiols thioredoxin (Trx) and Mycothiol (MSH) to reverse the oxidation of RsrA, restore thiol redox balance and re-establish the binding of RshA to SigH. The direct induction of *rshA* as a single transcript amplifies the shutdown of the SigH-dependent response after the cells have coped with the stress. The SigH regulon includes part of the SOS response and the heat-shock regulon, including the HspR and ClgR regulatory networks, which are responsible for protein quality control.

SigH is one of the major regulators, especially during heat stress, which also involves a number of different transcriptional regulators [8]. In contrast to studying the action of SigH in the presence of stress, we choose to uncouple SigH from RshA in order to assess its regulon without a possible stress-induced background. Using microarray analyses, we observed an induction of all SigH-dependent genes described by Ehira and coworkers in the *rshA* deletion mutant, with the exception of the *dnaK-grpE* operon, *clpC, sigB* and most genes of the *suf* cluster. Like Ehira and coworkers, working with overexpressing and deleting the *sigH* gene, we were unable to show a differential transcription of *clgR*. The rather weak transcriptional induction of some of the SigH-dependent heat-shock genes and the apparent absence of induction of the above-mentioned genes is explained by dominant effects exerted by known transcriptional regulators such as ClgR, HrcA, HspR, and/or SufR [14,16,18] (Figure 8). The additional action of these regulators might increase SigH activity under heat and/or oxidative stress. This might also hold for the *sigB* gene encoding the non-essential sigma factor of *C. glutamicum*. SigB is involved in gene expression in the transition phase of growth, and in our experiments sampling took place in the exponential phase of growth. Again, additional factors might be necessary to trigger the transcriptional activation of *sigB* by SigH.

Genes hitherto not described as being part of the SigH regulon included genes involved in mycothiol (MSH) synthesis and recycling. Besides thioredoxin (Trx), MSH is the major low-molecular mass thiol in corynebacteria, mycobacteria and streptomycetes [36]. The biosynthesis of MSH in *C. glutamicum* and two essential genes, *mshC* and *mshD* involved in the biosynthetic pathway have been described [37]. In our approach, we observed a SigH-dependent upregulation of *mshC*, coding for the second gene in mycothiol (MSH) synthesis, and *mca* as well as *mtr*, involved in mycothiol recycling (Figure 8).

*Mca* is the first gene in MSH recycling and was already shown to be transcribed in a SigH-dependent manner [14]. It encodes mycothiol *S*-conjugate amidase (Mca), which cleaves adducts (MSR) from the reaction of MSH with electrophiles to produce a mercapturic acid (AcCySR) and 1-*O-*(2-amino-2-deoxy-a-D-glucopyranosyl)-D-*myo*-inositol (GlcN-Ins) [30,31]. GlcN-Ins is the substrate of MshC, and MSH is synthesized from the subsequent enzymatic reaction with MshD [37]. As was mentioned above, *mshD* was not observed to be transcribed in a SigH-dependent manner, but its transcription was induced by disulphide treatment in *C. glutamicum* (our unpublished results), indicating that *mshD* is transcriptionally regulated. In *M*. *tuberculosis*, all the genes of MSH synthesis seem to be transcribed constitutively [30]. There is a similar mechanism in *S. coelicolor*, with the difference that besides *mca*, *mshA* is transcriptionally induced as a direct target of SigR and the genes *mshB, mshC* and *mshD* are SigR-dependent, but apparently induced indirectly [38].

The SOS regulon of many bacteria, including *E. coli*, is involved in various cellular processes, e.g. nucleotide excision and recombination repair [39]. By deleting the gene encoding the SOS response regulator LexA in *C. glutamicum*, Jochmann and coworkers [27] defined the SOS response in *C. glutamicum*, with only one of the *uvr* genes, namely *uvrC,* showing up in the microarray as differentially transcribed.

In our approach we observed a SigH-dependent induction of three *uvr* genes (*uvrA*, *uvrC, uvrD3*). The induction of *uvrC* transcription was quite low in our experiments, most likely because of an additional repression by LexA. As mentioned in [27], the degree of induction of SOS gene expression depends on at least four parameters: (i) the affinity of LexA for the SOS box, (ii) the location of the SOS box relative to the promoter, (iii) the promoter strength, and (iv) the presence of any additional constitutive promoters [39-41]. In this context, it is apparent that SigH is involved in the SOS response in *C. glutamicum*, integrating it with the heat stress and thiol-oxidative stress defense systems into a general stress response network.

This is in accordance with a proposal made by Barreiro *et al.*[18]. The regulation of *sigH* in cases of severe stress (probably causing DNA damage) would release LexA from the SOS boxes and thereby activate an additional SigA-dependent *sigH* promoter.

The SigH regulatory network appears to also control other functions. An interesting novel finding was the enhanced transcription of components of the proteasome. The actinobacterial proteasome consists of functions for pupylation (a process similar to eukaryotic ubiquitinylation, which marks proteins that are to be degraded) and proteases. Our study connects the recently identified pupylation component Pup (prokaryotic ubiquitin-like protein) and PafA2 (proteasome acessory factor, responsible for Pup conjugation; [14]) with the SigH regulon and underlines that SigH also plays a significant role in protein quality control.

Based on the results obtained in this study and in previous studies, we propose an extended model of the SigH regulon in *C. glutamicum* (Figure 8) including the direct control of the stress reponse to disulphide and heat stress by RshA, involving the thioredoxin system and the mycothiol-recycling system to cope with thiol-depleting conditions. In an unstressed state, SigH is inhibited by the reduced form of RshA. The disruption of the SigH–RshA complex in *C. glutamicum* appears under severe heat shock or disulphide stress via a change in the conformation through the oxidation of RshA. The released SigH forms a functional RNAP holoenzyme with the core enzyme and induces the stress response by transcribing SigH-dependent genes, including those involved in disulphide and heat stress response. The feed-forward induction of the anti-sigma factor RshA enables the cell to quickly shut down the stress response, based on SigH-dependent transcription, after the stress ends. RshA, as the stress-sensing redox switch, is one of the targets of the biochemical pathways encoded by genes of the SigH network, namely those of the reducing compounds thioredoxin (Trx) and mycothiol (MSH). Direct induction of *trxB1C* generates the thiol Trx and the gene products of *trxB*, *mtr*, *mca*, and *mshC* reduce and/or recycle Trx and MSH, respectively, which are able to restore, together with other reductases and reducing compounds, the thiol redox balance and reverse the oxidation of cysteine residues in RsrA. In this closed loop, RshA is reduced to regain its functionality and binds SigH after redox homeostasis is reached. A similar model was developed for the thiol-depleting stress response in *S. coelicolor* by Newton and coworkers in 2008 [30,31]. The transcriptional regulatory network controlled by SigH is highly connected to other regulators, modulating gene expression in response to other physical or chemical triggers. The heat-shock regulatory network that includes the regulators HspR and ClgR is an example of such a level of control.

## Conclusions

In this study, we approached the SigH regulatory network in *C. glutamicum* from another angle. In the absence of stress, the SigH regulon was induced by removing its cognate anti-sigma factor RshA. Our findings on the regulatory network on the one hand extended the known functions controlled by SigH, and on the other hand demonstrated that stress most likely imposes further actions that modulate the transcriptional control of apparently stress-related or unrelated genes. In the end, sigma factor competition at the RNAP determines whether an effect on the transcription of a certain gene is exerted as well as how strong it will be. In addition, factors like RNA degradation and proteolysis will surely have significant influences on all aspects of the network. Hence, a considerable amount of work lies ahead before we can claim that a single sigma factor network in *C. glutamicum* is understood.

## Methods

### Bacterial strains, plasmids, oligonucleotide primers, media and growth conditions

Bacterial strains and plasmids are listed in Table 2. Oligonucleotide primers are listed in Supplemental file 1. *E. coli* was cultivated in LB medium at 37°C, *C. glutamicum* was grown in complete 2xTY medium [42] or in minimal CGXII medium [43] containing protocatechuic acid (30 mg·l^-1^) in non-baffled shaking flasks at 30°C. When appropriate, nalidixic acid (50 *μ*g/ml for corynebacteria) or kanamycin (20 *μ*g/ml for *C. glutamicum* and 50 *μ*g/ml for *E. coli*) were added to the media.

**Table 2:** Plasmids and bacteria used in this work

**Plasmids**	**Relevant genotype/information**	**Source/reference**
pK18*mobsacB*	*sacB*, *lacZ*α, mcs (Km^R^)	[49]
pET2	*E. coli*–*C. glutamicum* promoter-probe vector (Km^R^, promoterless *cat* gene)	[58]
pET2*sigH*	*sigH* promoter region (550 bp) in pET2	this work
pET2*rshA*	*rshA* promoter region (301) in pET2	this work
pET2*sigH4*	P4*sigH* promoter region (348 bp) in pET2	this work
**Bacteria**		
***E. coli***		
*E. coli* JM109	*end*A1, *rec*A1, *gyr*A96, *thi*, *hsd*R17 (r_k_^–^, m_k_^+^), *rel*A1, *sup*E44, Δ(*lac-proAB*), F´ *tra*D36, *proAB*, *laqI*^*q*^*lacZ*ΔM15	[59]
***C. glutamicum***		
RES167	restriction-deficient *C. glutamicum* strain ( Δ*cglIM-cglIR-cglIIR*)	[47]
DN2	RES167 deletion of *sigH*	[24]
AS1	RES167 deletion of *sigH*-*rshA*	this work
Δ*rshA*	RES167 deletion of *rshA*	this work

### DNA isolation, manipulation and transfer

Isolation of plasmid DNA from *E. coli* cells by an alkaline lysis technique was performed using a QIAprep Spin Miniprep Kit (Qiagen, Hilden, Germany). Chromosomal *C. glutamicum* DNA was isolated as described previously [44]. DNA amplification by PCR was carried out with KOD DNA polymerase (Merck, Darmstadt, Germany) or Phusion DNA polymerase (Finnzymes,Vantaa, Finland) and chromosomal *C. glutamicum* RES167 DNA as the template. PCR products were purified with a QIAquick PCR Purification Kit (Qiagen). All oligonucleotides used in this study (Additional file 1) were purchased from Metabion (Martinsried, Germany). All PCR setups were done according to the manufacturers´ protocols. Modification of DNA, analysis by agarose gel electrophoresis and ligation were performed using standard procedures [42]. *E. coli* was transformed with plasmid DNA using the method of Hanahan [45], *C. glutamicum* cells were transformed by electroporation [46,47].

### Construction of defined deletions in the *C. glutamicum* chromosome

The defined chromosomal deletions (Δ*rshA*, Δ*sigH* and Δ*sigHrshA*) were constructed in *C. glutamicum* RES167 using the gene SOEing procedure [48], the *E. coli* vector pK18*mobsacB*[49] and the conditional lethal effect of the *sacB* gene for selecting double recombinants after the transformation of *C. glutamicum*[49]. The selection of the resulting marker-less *C. glutamicum* strains Δ*rshA*, DN2 and AS1 and PCR confimation of the respective *rshA* (220 bp) *sigH* (450 bp) and *sigHrshA* (1340 bp) deletions within their chromosomes (Figure 1A) were carried out as described previously [50] using the primers listed in the Additional file 3.

### Construction of plasmids

Fragments carrying the promoter regions of the genes *sigH, sigH*(P4), *rshA, mshC*, *mca, dnaJ2 uvrA* and *uvrD* were amplified from the chromosomal DNA of *C. glutamicum* by PCR with the primer pairs PSIGHF+PSIGHR, PSIGHF+PSIGH4R, PRSHAF+PRSHAR, PMSHCF+PMSHCR, PMCAF+PMCAR, PDNAJ2F+PDNAJ2R, PUVRAF+PUVRAR and PUVRDF+PUVRDR, respectively (Additional file 3). The primers carried the PstI, BamHI or BglII restriction sites. The PCR products were digested by the respective enzymes and cloned in the plasmid pET2 digested by PstI and BamHI. The resulting plasmid constructs were introduced into *C. glutamicum* by electroporation.

### RNA isolation and quantitative real-time RT-PCR

RNA was isolated from exponentially growing cultures of both *C. glutamicum* RES167 and the Δ*rshA* strain grown in triplicate. The cells were harvested by centrifugation and the cell pellets were immediately frozen in liquid nitrogen. The cells were then resuspended in the RLT buffer provided with the RNeasy Mini Kit (Qiagen, Hilden, Germany) and disrupted with a Precellys 24 homogeniser (Bertin Technologies, France) at a speed of 6.5 for 30 s once.

Total RNA was purified with an RNeasy Mini Kit along with an RNase-Free DNase Set (Qiagen) and a DNase I Kit (Sigma-Aldrich, Taufkirchen, Germany) according to a previously published protocol [34]. RNA was quantified with a NanoDrop ND-1000 spectrophotometer (Thermo Scientific, Wilmington, DE). Purified total RNA from *C. glutamicum* cultures was used in real time RT-PCR analysis performed with a LightCycler instrument (Roche Diagnostics, Mannheim, Germany) and a 2× SensiMix One Step Kit (Bioline, Luckenwalde, Germany). The verification of the resulting RT-PCR products was performed by melting curve analysis. The differences in gene expression were determined by comparing the crossing points of three samples measured in duplicate. The crossing points were determined using the LightCycler software (Roche Diagnostics). The calculation of the average crossing point (CP) was performed by first calculating the averages for each set of technical replicates and then by calculating the average of the three biological replicates. For each set of three biological replicates, the standard deviation was calculated (assuming a normal distribution of the CPs) and the combined standard deviation for the DeltaCP was approximated using the standard calculation for the propagation of uncertainity (assuming non-correlated errors).

### Microarray hybridization

The hybridization of whole-genome oligonucleotide microarrays was performed as described previously [51], using 8 μg of total RNA from *C. glutamicum* cultures for cDNA synthesis. The normalization and evaluation of the hybridization data was done with the software package EMMA 2 [52] using a signal intensity (*A*-value) cut-off of ≥7.0 and a signal intensity ratio (*M*-value) cut-off of ±0.6, which corresponds to relative expression changes equal to or greater than 1.5-fold.

### Northern blot analysis

The DIG-labeled RNA probes for the *sigH* and *rshA* genes for transcript analysis were obtained by *in vitro*-transcription with T7 RNA polymerase, NTP-DIG-label mix (Roche Diagnostics) and gene-specific primers with a T7 promoter-sequence attached to the reverse primer (Additional file 3). Prior to hybridization, the probes were denatured by incubation at 95°C for 10 min.

Northern blot analysis was performed as described by Homuth *et al.*[53] with the following modifications. Total RNA samples (5 μg), purified by using the RNeasy Mini Kit along with the RNase-Free DNase Set (Qiagen) and the DNase I Kit (Sigma-Aldrich) according to a previously published protocol [34], were separated under denaturing conditions in 1% agarose-formaldehyde gels in 1xMOPS (morpholinepropanesulfonic acid) running buffer and stained with ethidium bromide. Separated RNA was transferred to a Hybond-N membrane (GE Healthcare, Freiburg, Germany) by vacuum blotting. Hybridization and detection were carried out as follows. After being baked at 120°C for 0.5 h, the membrane was prehybridized under stringent conditions at 68°C for 1 h in 50% formamide and 5x SSC (1x SSC is 0.15 M NaCl plus 0.015 M sodium citrate) without the probe to block reactive membrane binding sites, and in the second step hybridized with digoxigenin (DIG)-labelled RNA probes (50 ng/ml) at 68°C overnight. The hybridized membrane was washed to remove the hybridization solution, first twice for 10 min in 2× SSC-0.1% (wt/vol) sodium dodecyl sulfate (SDS) at room temperature and then three times for 15 min in 0.1× SSC-0.1% (wt/vol) SDS at 68°C, and hybridization signals were detected according to the manufacturer’s instructions (Roche Anti-Digoxigenin-AP, Fab fragments 2 μl and CDP-*Star*) with a Luminescent Image Analyzer LAS-3000 (Fujifilm Europe, Düsseldorf, Germany). The sizes of the detected signals were determined by comparing with the prior ethidium-bromide-stained High Range Marker (Fermentas, St. Leon-Roth, Germany), marked on the membrane.

### Primer extension analysis

*C. glutamicum* cells were cultivated in 2xTY medium at 30°C, harvested at OD_600_ = 3.5, and frozen at −70°C. The pellet was resuspended in distilled water and approximately 0.2 × 10^8^ cells were disintegrated with a FastPrep FP120 (BIO101) (6x20 s, speed 6.0) using glass beads. The suspension was cooled for 5 min on ice between runs. The cell debris was removed by centrifugation and total RNA was isolated from the extract using a High Pure RNA Isolation Kit (Roche Diagnostics). The primer extension analysis was essentially done as described previously [54]. Reverse transcription was performed with SuperScript III transcriptase (Invitrogen, Carlsbad, CA) using 30 μg RNA and 5 pmol Cy-5-labeled primer CM4 or CM5 (Additional file 3) complementary to the vector pET2. Specific Cy5-labeled primers XMSHC, XMCA and XUVRD (Additional file 3) were used to determine the transcriptional start points of the genes *mshC*, *mca* and *uvrD*, respectively. PAA gel electrophoresis was run with the synthesized cDNA simultaneously with the DNA sequencing products generated with the same labeled primer in an A.L.F. Sequencer (GE Healthcare, Munich, Germany).

### Chloramphenicol acetyltransferase (CAT) assay

The CAT activity was essentially measured as described previously [55]. *C. glutamicum* strains harboring the vector pET2 with promoter-carrying fragments were cultivated in complete 2xTY medium to OD_600_ = 3 to 3.5. The cells were rapidly chilled on ice and disrupted with a FastPrep FP120 homogenizer (BIO101) (Thermo Scientific). The specific CAT activity in the cell-free extracts was determined photometrically at 412 nm as described by Shaw [56]. One unit (U) of enzyme activity was defined as 1 μmol of chloramphenicol acetylated per minute.

## Competing interests

The authors declare that they have no competing interests.

## Authors’ contributions

TB constructed the mutant, carried out the microarray analyses, Northern blots, and q-RT-PCR experiments, and drafted the manuscript. RS cloned the promoters, performed CAT assays, primer extension analyses and RACE experiments; MaP carried out cloning and performed primer extensions. MiP designed and evaluated the experiments of the Prague group and worked on the manuscript, JK conceived the study and finalized the manuscript. All authors read and approved the manuscript.

## Supplementary Material

Additional file 1 **Amino acid sequence alignment between the three corynebacterial genes and their *M*. *tuberculosis* and *S. coelicolor* counterparts**. Alignment of RshA from *C. glutamicum, C. efficiens, C. diphtheriae* and *C. jeikeium*, as well as *M*. *tuberculosis* and RsrA of *Streptomyces coelicolor* by CLUSTALX [60] Conserved cysteines are boxed. Identical residues are indicated with an asterisk, ":" indicates a stronger degree of conservation, and "." indicates a weaker degree of conservation. Click here for file

Additional file 2 **Genes differentially transcribed in *C***. ***glutamicum *** Δ***rshA *compared to *C***. ***glutamicum *RES167 (reference) sorted by ratio. **A comparative microarray hybridization analyses was performed using total RNA isolated from *C. glutamicum* RES167 [47] and its *rshA* deletion derivative growing under standard cultivation conditions (30°C) in shaking flasks. The differentially transcribed genes are listed in this table. Altogether, 83 genes in 61 putative transcriptional units were found to be upregulated in the Δ*rshA* mutant compared to its parental strain and 38 genes were downregulated, only 7 exceeded the chosen threshold m < −1 (fold change 2/0.5). Microsoft Excel. Click here for file

Additional file 3 **Primers used in this work. **Microsoft Word. Click here for file
