# Supplementary figures and images for: Transcriptional regulation of the operon encoding stress-responsive ECF sigma factor SigH and its anti-sigma factor RshA, and control of its regulatory network in Corynebacterium glutamicum
Source: BMC Genomics. 2012 Sep 3;13:445. doi: 10.1186/1471-2164-13-445 (PMC3489674; doi:10.1186/1471-2164-13-445)

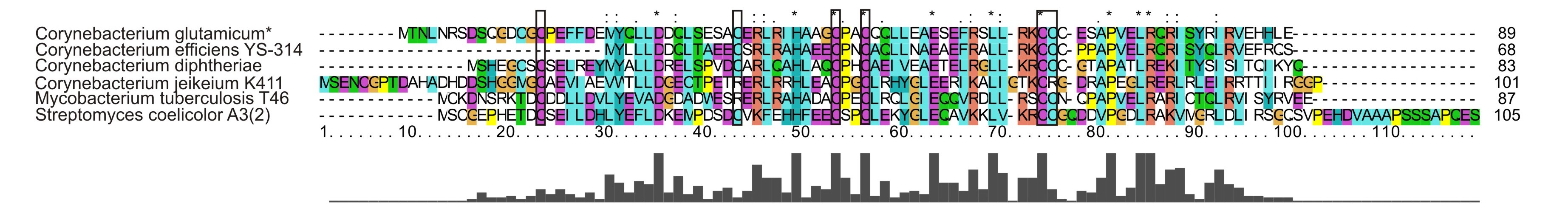

Supplement: Additional file 1 — Amino acid sequence alignment between the three corynebacterial genes and their M. tuberculosis and S. coelicolor counterparts. Alignment of RshA from C. glutamicum, C. efficiens, C. diphtheriae and C. jeikeium, as well as M. tuberculosis and RsrA of Streptomyces coelicolor by CLUSTALX [60] Conserved cysteines are boxed. Identical residues are indicated with an asterisk, ":" indicates a stronger degree of conservation, and "." indicates a weaker degree of conservation. [file 1471-2164-13-445-S1.jpeg]
