# Supplementary material for: Transcriptional regulation of the operon encoding stress-responsive ECF sigma factor SigH and its anti-sigma factor RshA, and control of its regulatory network in Corynebacterium glutamicum
Source: BMC Genomics. 2012 Sep 3;13:445. doi: 10.1186/1471-2164-13-445 (PMC3489674; doi:10.1186/1471-2164-13-445)
Supplement: Additional file 3 — Primers used in this work. Microsoft Word. [file 1471-2164-13-445-S3.doc]

**Additonal file 3**

Oligonucleotide primers used in this work

| **Primer** | **DNA sequence^a^** | | | | |  | | | **Properties*** | |
| --- | --- | --- | --- | --- | --- | --- | --- | --- | --- | --- |
|  | **Construction of chromosomal deletions** | | | | |  | | |  | |
| rshA_del1 | GATCTA*GAATTC*-AGGCACTGCCACTCCTTGAC | | | | |  | | | *Eco*RI | |
| rshA_del2 | CAGATGATGCTCGACACGAA-GAGTCGCTGCGGTTGAGATT | | | | | | |  | | |
| rshA_del3 | CGATGACGAATCTCAACCGC-TTCGTGTCGAGCATCATCTG | | |  |  | |  | | |  |
| rshA_del4 | GATCTATCTAGA-CAGGCCACTAGATGGTCTCC | | | | |  | | | XbaI | |
|  |  | | |  |  | |  | | |  |
|  | **Cloning promoters in pET2** | | |  |  | |  | | |  |
| PSIGHF | gaacttttt*ctgcag*ctttagtgat | | | | | | | PstI | | |
| PSIGHR | caacgc*agatct*atcgactgtgcc | | | | | | | BglII | | |
| PSIGH4R | cc*agatct*ccaccacagcagttagtaca | | | | | | | BglII | | |
| PRSHAF | aa*ctgcag*atgcaatgaatcaactc | | | | | | | PstI | | |
| PRSHAR | ga*ggatcc*cagggcagccacaatc | | | | | | | BamHI | | |
| PDNAJ2F | ac*ctgcag*ttacggctcccag | | | | | | | PstI | | |
| PDNAJ2R | gcctttt*ggatcc*ctgattcggtt | | | | | | | BamHI | | |
| PUVRAF | at*ctgcag*catcaatgagta | | | | | | | PstI | | |
| PUVRAR | tc*ggatcc*ttttaggttatgttcacg | | | | | | | BamHI | | |
| PMSHCF | tg*atgcat*agcgtgaagtagccacca | | | |  | |  | | | NsiI |
| PMSHCR | gg*ggatcc*agcgagagcagg | | | |  | |  | | | BamHI |
| PMCAF | caacttc*atgcat*gaaggtgtcgtcg | | | |  | |  | | | NsiI |
| PMCAR | tatt*ggatcc*atgatagtcgtttc | | | |  | |  | | | BamHI |
| PUVRDF | gt*ctgcag*aagaagctgaagggc | | | |  | |  | | | PstI |
| PUVRDR | ct*ggatcc*ggctaattgctgtggtga | | | |  | |  | | | BamHI |
|  |  | | | |  | |  | | |  |
|  |  | | | |  | |  | | |  |
|  | **Northern hybridization** | | | |  | |  | | |  |
| sigH_Northern1 | GCGGTGCTCTGCGCATGACTAGAA | | | | |  | | |  | |
| sigH_Northern2 | GAAATTAATACGACTCACTATAGG-GAGCCTCAACCTCGGCGGATTCCA | | | | | | | T7-Promoter sequence | | |
| rshA_Northern1 | GCGAAGGAACAAGGCATTGGTCTTGA | | | | |  | | |  | |
| rshA_Northern2 | GAAATTAATACGACTCACTATAGG-GTGCCGATTCGCAGCAGCACTT | | | | | | | T7-Promoter sequence | | |
|  |  | | |  |  | |  | | |  |
|  | **Primer extension** | | |  |  | |  | | |  |
| CM4 | GAAAATCTCGTCGAAGCGTCG | | | | |  | | | Cy5-labeled | |
| CM5 | aagctcggcggatttgtc | | |  |  | |  | | | Cy5-labeled |
| XMSHC | CGACAAGCTATTTGCAAGCTAT | | |  |  | |  | | | Cy5-labeled |
| XMCA | CTGAGGTGACTAAGATGTA | | |  |  | |  | | | Cy5-labeled |
| XUVRD | GAGACCTCCTGTTTCCTGAT | | |  |  | |  | | | Cy5-labeled |
|  |  | | |  |  | |  | | |  |
| **Primer** | **DNA sequence^a^** | | | | |  | | | **Properties*** | |
|  | **Q-RT-PCR** | | |  |  | |  | | |  |
| arnA_LC1 | | GCTGCGGACATAGTATGTTC |  | | | | | | | |
| arnA_LC2 | | ACATCGTTACCCCCGAACAG |  | | | | | | | |
| cg2838_LC1 | | CAGCACATCGAGGTCATCAA |  | | | | | | | |
| cg2838_LC2 | | AATGAACGGCCAAGTCCAAG |  | | | | | | | |
| cg1560_uvrA_LC1 | | AACCAGCGCTTGATCACTAC |  | | | | | | | |
| cg1560_uvrA_LC2 | | GACAAGTAAGCACCTGTGAG |  | | | | | | | |
| cg2194_mtr_LC1 | | TCACCGCAGTACACAACAAG |  | | | | | | | |
| cg2194_mtr_LC2 | | ACTGGTGCGACCGAATTCAT |  | | | | | | | |
| cg1709_mshC_LC1 | | TGGCTACGAGTCCAATTACG |  | | | | | | | |
| cg1709_mshC_LC2 | | GTTGGTAGCGATTGCTGAAC |  | | | | | | | |
| cg1555_uvrD_LC1 | | AATCATCGTGCAGCTGATCG |  | | | | | | | |
| cg1555_uvrD_LC2 | | ATCGTGGTCTCTGTTGATGG |  | | | | | | | |
| cg2838_LC1 | | CAGCACATCGAGGTCATCAA |  | | | | | | | |
| cg2838_LC2 | | AATGAACGGCCAAGTCCAAG |  | | | | | | | |
| cg3405_LC1 | | CGCCATCAGCCTTCTTGATT |  | | | | | | | |
| cg3405_LC2 | | TTGAGCAGCGAGTTCTTGGA |  | | | | | | | |
| cg1127_mca_LC1 | | CGGATCACCTCAAGGTTCAT |  | | | | | | | |
| cg1127_mca_LC2 | | AGCCATCACATCAGCCTCAT |  | | | | | | | |
| cg0876_sigH_LC1 | | TCAAGGCGTACCAGGCGTTC |  | | | | | | | |
| cg0876_sigH_LC2 | | TCAACCTCGGCGGATTCCAG |  | | | | | | | |
